# On-surface synthesis of disilabenzene-bridged covalent organic frameworks

**DOI:** 10.1038/s41557-022-01071-3

**Published:** 2022-11-07

**Authors:** Kewei Sun, Orlando J. Silveira, Yujing Ma, Yuri Hasegawa, Michio Matsumoto, Satoshi Kera, Ondřej Krejčí, Adam S. Foster, Shigeki Kawai

**Affiliations:** 1 Research Center for Advanced Measurement and Characterization, National Institute for Materials Science, Tsukuba, Japan; 2 Department of Applied Physics, Aalto University, Espoo, Finland; 3 Department of Photo-Molecular Science, Institute for Molecular Science, Okazaki, Japan; 4 International Center for Materials Nanoarchitectonics (WPI-MANA), National Institute for Materials Science, Tsukuba, Japan; 5 WPI Nano Life Science Institute (WPI-NanoLSI), Kanazawa University, Kakuma-machi, Japan; 6 Graduate School of Pure and Applied Sciences, University of Tsukuba, Tsukuba, Japan

**Keywords:** Scanning probe microscopy, Molecular self-assembly, Surface assembly, Materials chemistry

## Abstract

Substituting carbon with silicon in organic molecules and materials has long been an attractive way to modify their electronic structure and properties. Silicon-doped graphene-based materials are known to exhibit exotic properties, yet conjugated organic materials with atomically precise Si substitution have remained difficult to prepare. Here we present the on-surface synthesis of one- and two-dimensional covalent organic frameworks whose backbones contain 1,4-disilabenzene (C_4_Si_2_) linkers. Silicon atoms were first deposited on a Au(111) surface, forming a AuSi_*x*_ film on annealing. The subsequent deposition and annealing of a bromo-substituted polyaromatic hydrocarbon precursor (triphenylene or pyrene) on this surface led to the formation of the C_4_Si_2_-bridged networks, which were characterized by a combination of high-resolution scanning tunnelling microscopy and photoelectron spectroscopy supported by density functional theory calculations. Each Si in a hexagonal C_4_Si_2_ ring was found to be covalently linked to one terminal Br atom. For the linear structure obtained with the pyrene-based precursor, the C_4_Si_2_ rings were converted into C_4_Si pentagonal siloles by further annealing.

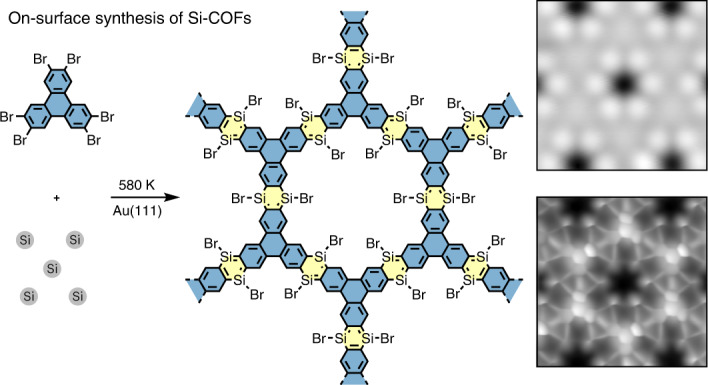

## Abstract (web summary)

Incorporating silicon into organic molecules and materials leads to interesting changes in electronic structure and properties; silabenzenes are attractive species for this purpose, but their high reactivity in solution poses challenges. Now, 1D and 2D covalent organic frameworks featuring disilabenzene rings (C_4_Si_2_) as linkers have been prepared by reacting silicon atoms and polyaromatic hydrocarbon precursors on a Au(111) surface.

## Main

Covalent organic frameworks (COFs), as a large class of porous organic materials, have attracted intense research in the past few decades due to the great potential for applications in the fields of, for example, catalysis^1,2^, optoelectronics^3,4^ and gas storage and separation^5,6^. Since the seminal work on COF synthesis by Yaghi and co-workers in 2005 (ref. ^7^), developments in both solvothermal and on-surface syntheses have increased the diversity of COF structures, which are mainly composed of light elements (B, C, N, O, H). COF syntheses employed Schiff base reactions and self-condensation of boronic acid, as well as coupling between boronic acid and catechol^1,7–10^. More recent efforts introducing new chemical reactions connecting aromatic multi-substituted monomers have succeeded in the synthesis of conjugated two-dimensional COFs (refs.^11,12^). If the heavy elements are substituted in the precursor molecules, the elemental variety is drastically enhanced^13^.

Silicon is an element of group 14 in the periodic table. Its four valence electrons at the outermost shell give it similar properties to carbon, yet the longer bond lengths and possible higher bonding states lead to a higher Lewis acidity and unique chemical reactivities^14,15^. Silicon-incorporated organic functional molecules and novel nanostructures have attracted much attention in recent decades^16,17^. For instance, silicates (containing Si–O bonds) are some of the most studied compounds^18,19^ and the successful synthesis of the silicate-COF was recently demonstrated^20^. The silyl group, commonly used as a protecting group in solution^21^, was found to induce coupling reactions on surfaces^22–25^. By contrast silabenzenes, having unique heterocyclic rings with C–Si bonds, represent the heavier congeners of cyclic aromatic compounds in fundamental organic chemistry^26,27^. These have been studied as an elusive target product for organic synthesis due to their high reactivity at ambient temperatures and their difficult isolation^28–30^. As yet, only a few silabenzene compounds have been reported in solution^28–35^. Silabenzenes are very promising candidates in on-surface synthesis to create COFs and other silicon-incorporated nanostructures. In this Article, we present the on-surface synthesis of disilabenzene-bridged COFs by deposition of silicon atoms on a Au(111) surface and annealing followed by reaction with 2,3,6,7,10,11-hexabromotriphenylene (HBTP). Their structures and chemical properties are analysed using a combination of bond-resolved scanning tunnelling microscopy (STM), scanning tunnelling spectroscopy (STS), photoelectron spectroscopy and density functional theory (DFT) calculations. The synthesis of the conjugated COFs by on-surface coupling of Si atoms and polyaromatic hydrocarbons may pave the way for fabrication of novel low-dimensional nanostructures.

## Results and discussion

### Synthesis of 1,4-disilabenzene-linked conjugated COFs

We used on-surface synthesis to realize a silabenzene-bridged COF under ultra-high vacuum conditions^36,37^. This bottom-up method has proved successful in the synthesis of graphene nanoribbons (GNRs) with different edges, including different atomic species^38,39^. Once GNRs are merged with each other at their edges, wider GNRs or even two-dimensional COFs can be synthesized^40,41^. Alternatively, COFs have also been synthesized with predefined and small precursors^42,43^. However, in some cases, such as for the unstable silabenzene, no direct precursor is available.

**Fig. 1:**
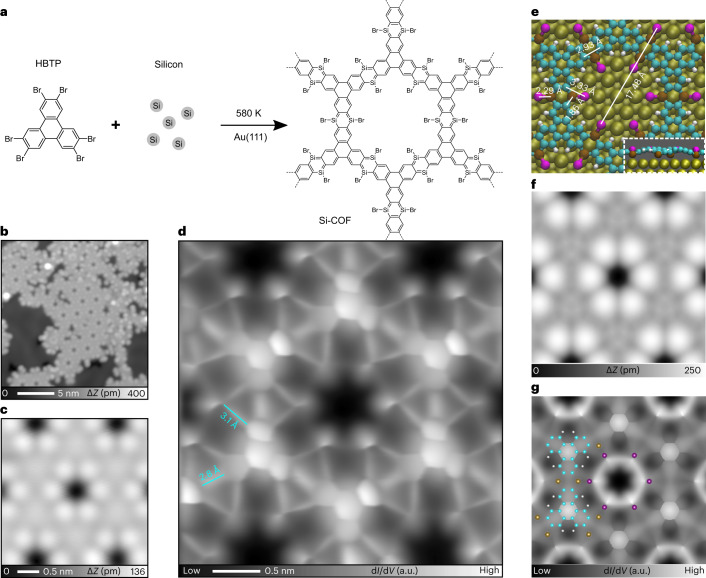
Synthesis of Si-incorporated COF. **a**, Scheme of on-surface reaction for the aryl–Si coupling reaction on Au(111). **b**, STM topography of the sample after annealing at 580 K. Sample bias voltage *V* = 100 mV and tunnelling current *I* = 20 pA. **c**, Close-up STM image of the Si-COF. *V* = 200 mV and *I* = 5 pA. **d**, High-resolution constant height d*I*/d*V* map of the Si-COF taken with a CO tip. a.u., arbitrary unit. **e**, Predicted top-view structure from DFT simulations with key bond lengths (Å) indicated. Inset, side view. Atom colour code: C, cyan; H, white; Au, gold; Si, brown; Br, purple. **f**,**g**, Associated simulated STM images showing STM topography at 200 mV and constant charge density of 1 × 10^−8^ e Å^−3^ (**f**) and high-resolution image at a bias of 0.5 V and a height of 0.3 nm (**g**).

**Extended Data Fig. 1:**
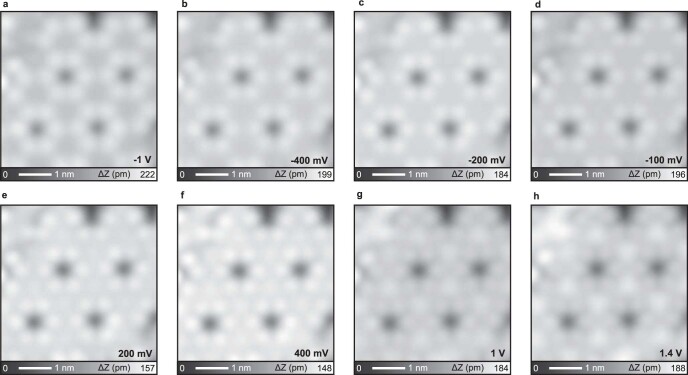
Si-COFs on Au(111). **a**–**h**, A series of STM topographies of Si-COFs measured with different bias voltages. The corresponding voltage values are labeled at the bottom right in each image.

**Extended Data Fig. 2:**
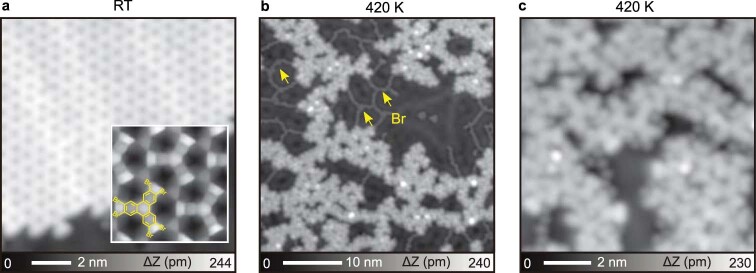
HBTP molecules on Au(111). **a**, STM topography of Au(111) surface kept at room temperature after depositing HBTP molecules. Inset shows the bond-resolution constant height STM image of close-up view. **b**, Typical STM image of Au(111) surface with HBTP molecules after annealing at 420 K for 10 minutes. Large scale irregular structures and scattered bromine atoms as indicated by arrows after debromination are seen. **c**, A close-up view of irregular nanostructures after reaction of HBTP molecules. These results demonstrate that it is difficult to form well-ordered COFs by only HBTP molecules on Au(111). Measurement parameters: *V* = 10 mV and *I* = 100 pA in (**a**). *V* = 200 mV and *I* = 20 pA in (**b**). *V* = 50 mV and *I* = 20 pA in (**c**).

**Extended Data Fig. 3:**
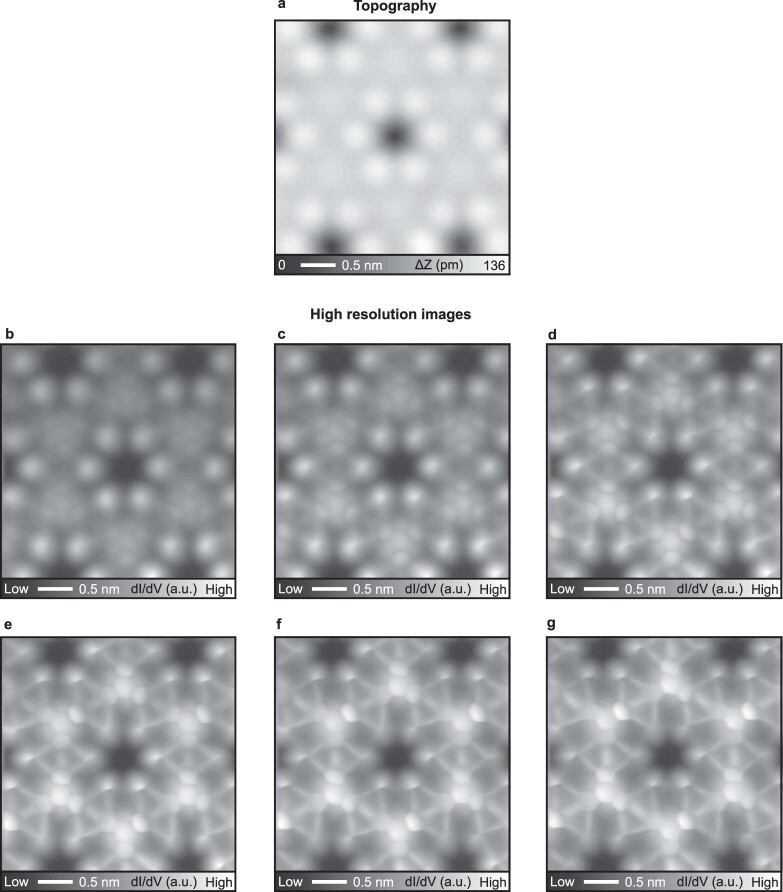
Bond-resolved images of Si-COF. **a**, STM topography of the detailed Si-COF unit. **b**–**g**, A series of high-resolution constant height d*I*/d*V* images taken by a CO-tip with decreasing tip-sample distances. Measurement parameters: *V* = 200 mV and *I* = 5 pA in (**a**).

**Extended Data Fig. 4:**
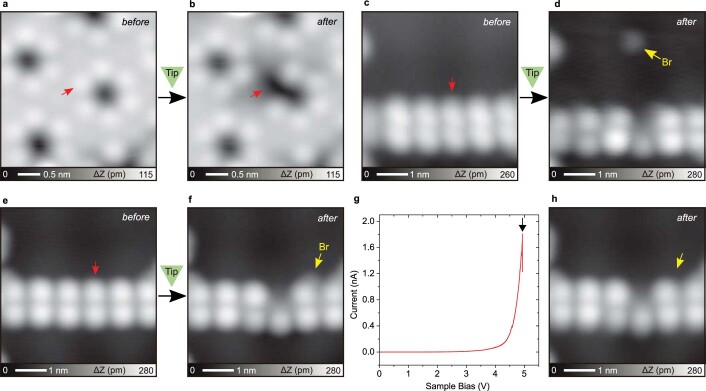
Tip-induced debromination. **a**, STM topography of Si-COF. A metal tip was positioned at the site indicated by an arrow. **b**, STM topography taken after applying a large sample bias of *V* = 3.6 V (*I* = 210 pA). The contrast around Site **1** became darker due to tip-induced debromination. **c**, STM topography of Si-Cove GNR. A metal tip was positioned at the site indicated by an arrow. **d**, STM topography taken after applying a bias voltage of 4.9 V. The contrast around Site **2** became darker. We found the dissociated Br atom as indicated by an arrow. **e**,**f**, Another example of the tip-induced debromination on Si-Cove GNR. **g**, *I*-*V* curve recorded during the debromination. An abrupt change of the tunneling current was detected when the bias voltage of 4.9 V was applied to the sample. The Br atom was attached to the edge of Si-Cove GNR as indicated by an arrow in (**f**). **h**, STM topography of Si-Cove GNR after tip picking up the dissociated Br atom by the tip. Measurement parameters: *V* = 50 mV and *I* = 20 pA in **a**,**b**. *V* = 200 mV and *I* = 10 pA in (**c**,**d**,**e**,**f**,**h**).

**Extended Data Fig. 5:**
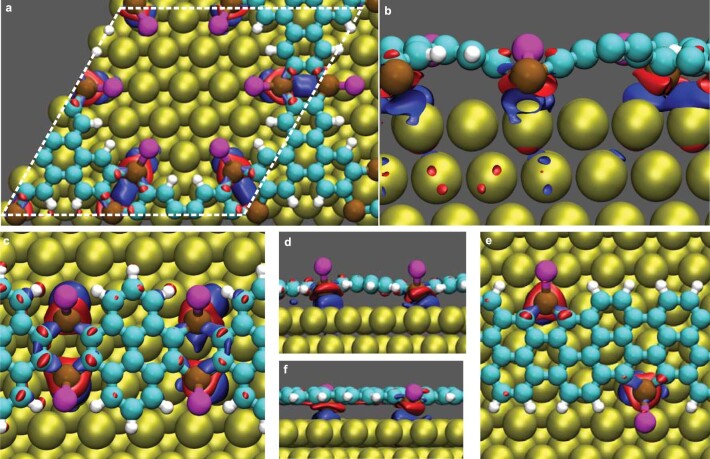
Calculated charge transfer between Si and Au atoms and adsorption heights of Br atoms for Si-COF. **a**–**f**, Calculated charge transfer between Si and Au atoms and adsorption heights of Br atoms for Si-COF: (**a**) top view, (**b**) side view, Si-Cove GNR: (**c**) top view, (**d**) side view, and Si-Armchair GNR: (**e**) top view, (**f**) side view. ±0.01 charge contours (blue accumulation, red depletion). Only minimal changes in bond lengths and charges for equivalent C, Si and Br sites when comparing the COF on Au with the relaxed, fully planar, system in isolation. On the other hand, the adsorption heights of Br atoms in the C_4_Si_2_ rings between the Si-COF and Si-Cove GNRs are different, which relates to the difference of the apparent Br atom sizes.

In this Article, we overcome this limitation by combining conventional surface science techniques and on-surface chemistry. We used HBTP as a building block to fabricate a two-dimensional Si-incorporated COF (Fig. 1a). Firstly, a submonolayer AuSi_*x*_ film was formed on Au(111) by depositing Si atoms at room temperature with a post-anneal to 420 K (Supplementary Fig. 1). HBTP molecules were deposited on the substrate held at 420 K, causing debromination. Consequently, the sample was further annealed at a higher temperature of 580 K. We found the formation of hexagonal nanoporous structures in the STM topography (Fig. 1b). The pores are surrounded by six bright spots, with equivalent sites separated by 1.75 ± 0.02 nm (Fig. 1c). The contrast of the nodal site and surrounding six bright spots changes with respect to the bias voltage (Extended Data Fig. 1). Since following a similar growth procedure method without pre-deposited Si atoms on a clean Au(111) surface resulted in formation of disordered films (Extended Data Fig. 2), we conclude that Si atoms play a decisive role in the synthesis of the structure. Before the final step of annealing at 580 K, we also found the formation of SiBr_*x*_ (*x* = 1, 2, 3) compounds on the Au terrace as well as on the nanoporous structure (Supplementary Fig. 2)—these can be desorbed as SiBr_4_ molecules from the surface by annealing at 450 K (ref. ^44^). To resolve the inner structures of the framework, the tip apex was terminated by a CO molecule^45,46^. The bond-resolved STM image taken at a constant height mode indicates that the structure was composed of six triphenylene backbones at the nodal site (Fig. 1d and Extended Data Fig. 3), which are inter-connected by two different types of bond. The length of the longer one (3.10 ± 0.20 Å) is far more than the typical length of a covalent bond, indicating that it is composed of more than two atoms. We tentatively assigned this longer line as C–Si–X (X = Br or H) bonds, where Br atoms are from HBTP molecules, while H atoms are possibly from the chamber environment. In our previous study, Si and Br atoms can easily form a covalent bond on Au(111) (Fig. 1a)^44^. The central bond between two neighbouring triphenylene backbones has a shorter length (2.80 ± 0.20 Å), which we suggest is due to deflection of the CO tip during scanning and is not a physical bond^47^. In order to investigate the structure behind these images, we undertook an extensive DFT analysis of possible molecular assemblies, including a wide variety of configurations and atoms in the network. The best agreement is shown in Fig. 1e, suggesting the Si atoms in the C_4_Si_2_ ring are passivated by Br atoms (a Si atom at the edge of the Si-COF can also bond to two Br atoms (Supplementary Fig. 3)). We see a very good agreement with experiment in the STM-simulated topography (Fig. 1f), with pores also separated by 1.75 nm. We also see good agreement in the comparison between the high-resolution CO-tip STM images—the simulated image in Fig. 1g reproduces the sharp contrast over the central triphenylene backbone and shows a thick line between neighbouring triphenylenes (2.93 Å) and also a long line over C–Si–Br (3.33 Å). Note that the Si–Br bond in the COF can be cleaved by applying a bias voltage (Extended Data Fig. 4). The analysis of induced charge-density differences shows the interaction between Si and Au stabilizing the COF on the substrate (Extended Data Fig. 5). However, we observe minimal changes in bond lengths and charges for equivalent C, Si and Br sites when comparing the COF on Au with the relaxed, fully planar, system in isolation, suggesting that the properties of the planar COF in isolation and the COF on Au are similar. This is further confirmed by a comparison of the nucleus-independent chemical shift (NICS) between characteristic molecules, the isolated COF and the COF on Au (Supplementary Figs. 4 and 5). Overall, this confirms that the triphenylene blocks are connected via the planar C_4_Si_2_ ring, resulting in 1,4-disilabenzene-linked COFs.

### Electronic properties of Si-COFs

**Fig. 2:**
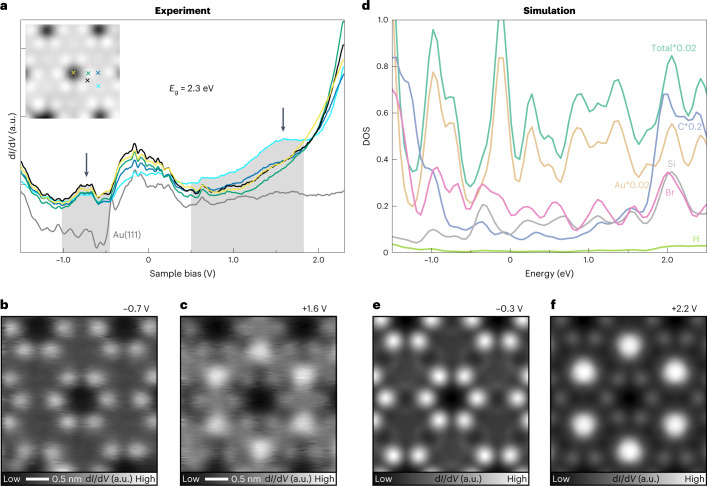
Electronic properties of Si-COF. **a**, d*I*/d*V* curves were recorded above the Si-COF (inset image) and the Au(111) surface for contrast. The corresponding sites are indicated by coloured crosses. a.u., arbitrary unit. **b**,**c**, Constant height d*I*/d*V* maps were measured with a CO-terminated tip at −0.7 V (**b**) and +1.6 V (**c**). **d**, Calculated density of states (DOS) for C, H, Si and Au as well as for the total of the atoms. **e**,**f**, Simulated constant height d*I*/d*V* maps of the Si-COF at biases of −0.3 V (**e**) and +2.2 V (**f**).

The electronic properties of the Si-COF were measured in more detail using STS (Fig. 2a). Due to the interaction with the Au substrate, the occupied state was detected only at the centre of the triphenylene block (light blue curve) and was significantly broadened as indicated by the grey areas. In fact, such a transition of the contrast can be clearly seen in a series of constant height d*I*/d*V* maps (Fig. 2b,c and Supplementary Figs. 6 and 7). The interaction between the Si-COF and Au(111) surface also leads to the broadening of the unoccupied state peaks, which are localized around the Br sites. Nevertheless, we assigned the unoccupied and occupied states as 1.6 and −0.7 V, resulting in a band gap of approximately 2.3 eV. On the other hand, the distinct electronic states of a Si atom incorporated in the Si-COF and a single Si atom adsorbed on a clean Au(111) surface were also measured by STS, indicating a different chemical nature (Supplementary Fig. 8). The calculated density of states (DOS) (Fig. 2d) shows similar features to the STS, with matching rapidly rising densities of states around −1.5 and 2.0 V. This rapid rise is associated with the states coming from the molecular backbone of the COF, as the contribution from the C atoms significantly increases, while the contributions from the Si and Br atoms remain more or less constant in the interval of energy shown in Fig. 2d. The direct comparison of the simulated occupied states image at −0.3 V (Fig. 2e) reproduces the experimental dominance of the Br atoms, with nothing seen on the molecular backbone. Supplementary Fig. 9 shows that the Br dominance is actually observed in several occupied states within the window of −1.0 to 0.0 V, and in the empty states as well, up to 0.8 V. However, for higher biases, contributions from both Br atoms and the molecular backbone are observed uniformly, until the general feature previously observed is inverted. Figure 2f, for example, shows this inversion at 2.2 V, where the strong dominance of the molecular backbone seen in the experiment is finally reproduced.

### Photoemission spectroscopy in each reaction step

**Fig. 3:**
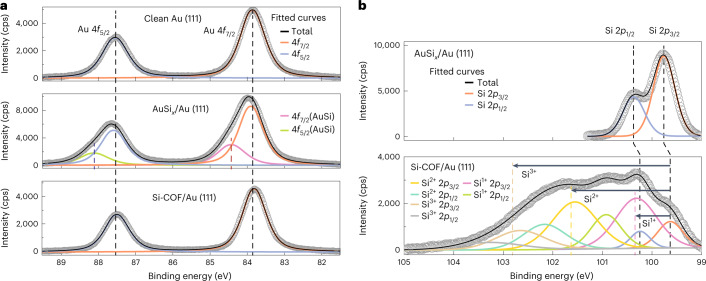
Photoemission spectroscopy measurement. **a**, Au 4*f* curves at each reaction step (top to bottom: clean Au(111) substrate, AuSi_*x*_/Au(111) and Si-COF/Au(111) surfaces). Au 4*f* spectra of clean Au(111) substrate show doublet peaks of Au 4*f*_7/2_ (83.8 eV) and Au 4*f*_5/2_ separated by 3.69 eV. Au 4*f* doublet peaks of AuSi_*x*_/Au(111) were broadened by other components with a higher binding energy (BE) of 0.51 eV. Au 4*f* spectra of Si-COF/Au(111) became comparable to those of clean Au(111). **b**, Si 2*p* core level spectra at each reaction step. Si 2*p* spectra of the AuSi_*x*_/Au(111) surface show the characteristic Si 2*p* doublet peaks with a small separation from spin–orbit coupling of *p* orbitals (0.61 eV) as the Si 2*p*_3/2_ peak is located at 99.75 eV. Si 2*p* spectra of the Si-COF/Au(111) surface have four sets of doublet peak components after fitting, showing three different charge states of the Si atom (those components are located 0.71, 2.0 and 3.18 eV higher in BE than elemental Si, respectively). Open circles in the plots denote the Shirley-background-subtracted XPS data.

To investigate the chemical properties of the Si-COF, we carried out synchrotron photoemission spectroscopy measurements after each reaction step. The clean surface of the Au(111) substrate was first ensured by the presence of well-defined spin–orbit doublet peaks of Au 4*f*_7/2_ (83.8 eV) and Au 4*f*_5/2_ separated by 3.69 eV. These energies are in excellent agreement with established numbers (Fig. 3a)^48–50^. After depositing Si atoms on a clean Au(111) surface, Au 4*f* doublet peaks (Fig. 3a) were significantly broadened by other components with a higher binding energy (BE) of 0.51 eV, which corresponds to the AuSi_*x*_ alloy (Supplementary Fig. 10)^51–53^. After formation of the Si-COF, the Au 4*f* spectra became comparable to those of clean Au 4*f* doublet peaks (Fig. 3a). As observed in the STM measurements, the dissociated Br atoms from HBTP molecules tend to react with Si atoms and consequently remove the AuSi_*x*_ layer from the surface by forming highly volatile SiBr_4_ molecules^34^. This phenomenon was also identified in the Br 3*d* spectra (Supplementary Fig. 11) where we observed a significant reduction in the signals of Br 3*d* after further annealing at a higher temperature.

The corresponding Si 2*p* spectra of the AuSi_*x*_ layer on Au(111) show the characteristic Si 2*p* doublet peaks with a small separation from spin–orbit coupling of *p* orbitals (0.61 eV) as the Si 2*p*_3/2_ peak is located at 99.75 eV (Fig. 3b and Supplementary Fig. 12). We assume that charge transfer from Si to Au is responsible for the small shift to higher energy, compared with a value measured in the bulk (99.3 eV)^54^. After the synthesis of the COF, the Si 2*p* spectra became complex. Our best fitting is consistent with four sets of doublet peak components, which can be associated to three different charge states of the Si atom (in our case, those components are located 0.71, 2.0 and 3.18 eV higher in BE than elemental Si, respectively). Although these numbers differ slightly from those of integer charge states of Si^1+^ (1 eV), Si^2+^ (1.81 eV) and Si^3+^ (2.63 eV) measured in bulk inorganic form^55,56^, the Si-COF exhibits two major species with relatively large peak area that are located at 0.71 and 2.0 eV higher BE than the elemental Si position. Since the component shifted by 2 eV is almost comparable to a Si^2+^ charge state (1.81 eV), it is reasonable to assign it with the proposed COF structure (Fig. 1a,d,e) where the Si atom is covalently bonded with two carbon atoms and a bromine atom. Therefore, as both carbon and bromine have higher electronegativities than Si, a slightly higher charge state than Si^2+^, but less than that of Si^3+^, can be expected for these C_4_Si_2_Br_2_ linker units. We attributed the other major component that is shifted by 0.71 eV in BE to the Si atoms that are incorporated on the edges of the Si-COF and are bonded with one carbon and one Br atom (Supplementary Fig. 3). The peak at 3.18 eV is from the Si at the edges, which is bonded with one carbon and two Br atoms (Supplementary Fig. 3).

### Thermal transformation of 1,4-disilabenzene to silole in a linear COF analogue

**Fig. 4:**
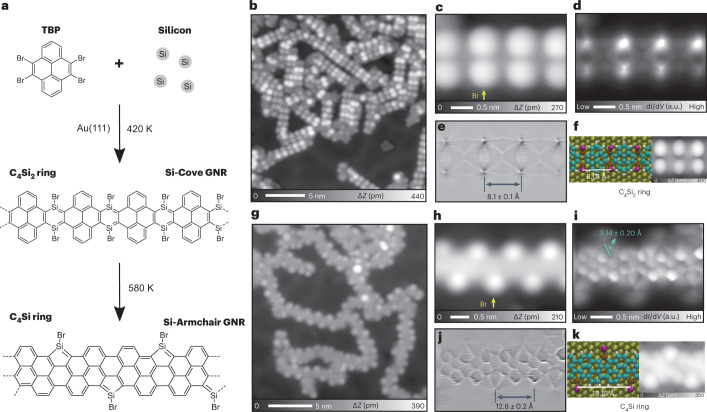
Synthesis of Si-doped GNRs. **a**, Scheme of on-surface synthesis of two types of Si-doped GNR on Au(111). **b**, STM topography of Si-covered Au(111) held at 420 K after depositing TBP molecules. Sample bias voltage *V* = 200 mV and tunnelling current *I* = 5 pA. **c**, Close-up view of Si-Cove GNR. *V* = 200 mV and *I* = 5 pA. **d**,**e**, Constant height d*I*/d*V* image of the area in **c** (**d**) and the corresponding Laplace filtered image (**e**). **f**, Simulated chemical structure of Si-Cove GNR and associated simulated STM image at 200 mV and constant charge density of 1 × 10^−8^ e Å^−3^. **g**, STM topography of sample after annealing at 580 K. *V* = 100 mV and *I* = 10 pA. **h**, Close-up view of Si-Armchair GNR. *V* = 100 mV and *I* = 20 pA. **i**,**j**, Constant height d*I*/d*V* image of the area in **h** (**i**) and the corresponding Laplace filtered image (**j**). **k**, Simulated chemical structure of Si-Armchair GNR and associated simulated STM image at 200 mV and constant charge density of 5 × 10^−8^ e Å^−3^.

**Extended Data Fig. 6:**
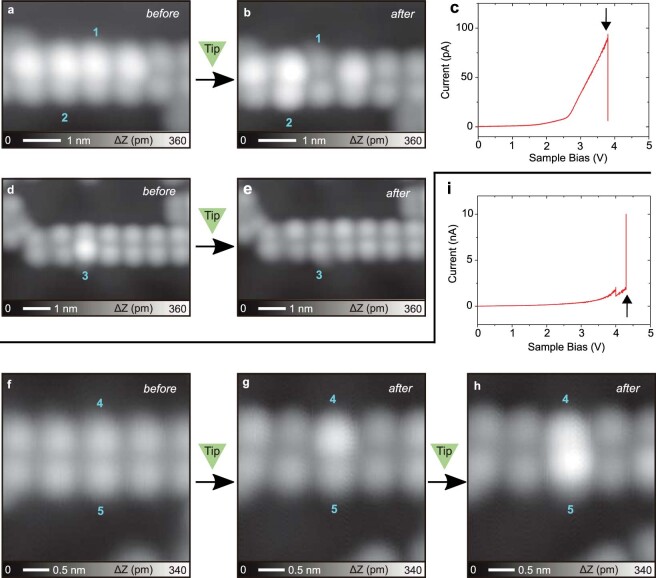
Tip-induced switch of the Br contrast in Si-Cove GNRs. **a**,**b**, STM topographies showing four brighter dots at the upper sites (**a**) and after sweeping the sample bias voltage (**b**). The contrast of Site 1 became darker (switch off). Consequently, the contrast of Site 2 became brighter (switch on). **c**, Recorded I-V curve during the switch. At *V* = 3.8 V, an abrupt change of the tunneling current was detected. **d**–**h**, Other examples of simple ‘switch off’ (**d**,**e**) and ‘switch on’ (**f**–**h**). **i**, Recorded *I*-*V* curve during the ‘switch on’. An abrupt increase of the tunneling current was measured at V = 4.3 V after the small reduction of the tunneling current at 3.9 V. Measurement parameters: *V* = 200 mV and *I* = 10 pA in (**a**,**b**). *V* = 200 mV and *I* = 20 pA in (**d**,**e**). *V* = 200 mV and *I* = 50 pA in (**f**,**g**,**h**).

**Extended Data Fig. 7:**
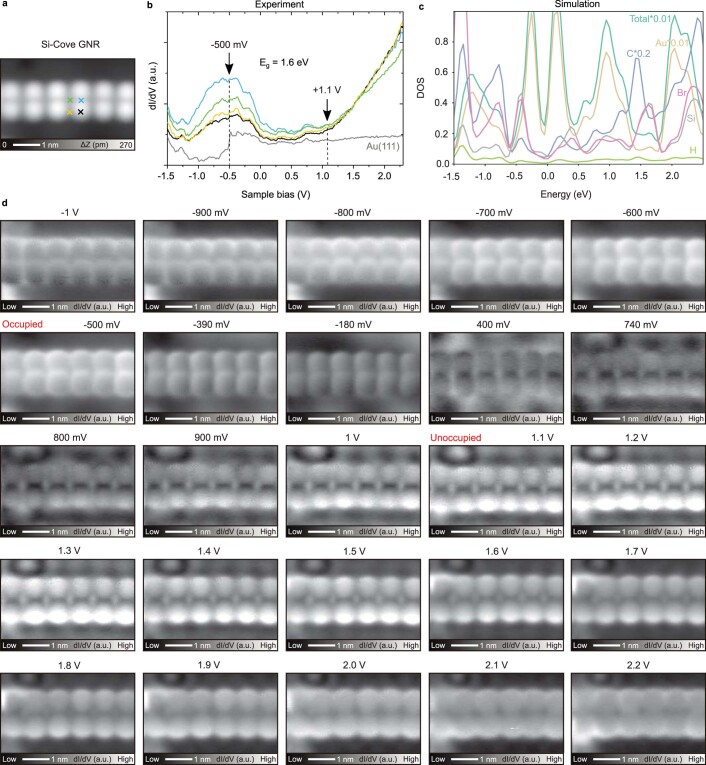
Electronic properties of Si-Cove GNR. **a**,**b**, Close-view STM topography (**a**) d*I*/d*V* curves (**b**) measured at the four different sites indicated by crosses in (**a**) as well as on Au(111) surface as a reference. **c**, Calculated Density of States (DOS). **d**, A series of constant current d*I*/d*V* maps measured at different bias voltages. We assigned the occupied state at around −0.5 V and the onset of unoccupied state (no peak visible) at around 1.1 V, resulting in a band gap of 1.6 eV. In the d*I*/d*V* maps, the occupied and unoccupied states are localized around the Br sites and the carbon backbone, respectively. Measurement parameters: *V* = 200 mV and *I* = 100 pA.

To demonstrate the generality of the Si–C bond formation by co-deposition of Si atoms and bromo-substituted molecules, we used 4,5,9,10-tetrabromopyrene (TBP) molecules, in which two groups of *ortho*-bromine atoms are introduced at both sides of the pyrene backbone (Fig. 4a). After TBP molecules were deposited on the partially covered AuSi_*x*_ layer on Au(111) held at 420 K, short oligomers appeared (Fig. 4b). The close-up view (Fig. 4c) shows that bright dots are located at the edges of the longitudinal axis. A constant height d*I*/d*V* image taken with a CO-terminated tip (Fig. 4d) and the corresponding Laplace filtered image for enhancement of bond features (Fig. 4e) show the detailed structures, in which the pyrene backbones are connected via disilabenzene. The bright dots indicated by arrows in Fig. 4c correspond to Br atoms. Comparison to the simulated structure and STM images (Fig. 4f) confirms that a Si-doped cove-edge graphene nanoribbon (Si-Cove GNR) was synthesized. As seen in Fig. 4b and Supplementary Fig. 13, the contrast of the Br atoms significantly varies. Since the tip-induced manipulation allowed iterative switches (Extended Data Fig. 6), the difference in adsorption height should be responsible for the difference in contrast, indicating a certain flexibility of the non-planar C_4_Si_2_ ring. Note that the difference of the adsorption heights of Br atoms in the C_4_Si_2_ rings between the Si-COF and Si-Cove GNR also relates to the difference of the apparent Br atom sizes (Extended Data Fig. 5). The electronic properties of Si-Cove GNR were investigated by STS measurement and DFT calculations (Extended Data Fig. 7). We determined that the occupied and unoccupied states were located at around −0.5 and + 1.1 V, respectively, resulting in a band gap of 1.6 eV.

**Extended Data Fig. 8:**
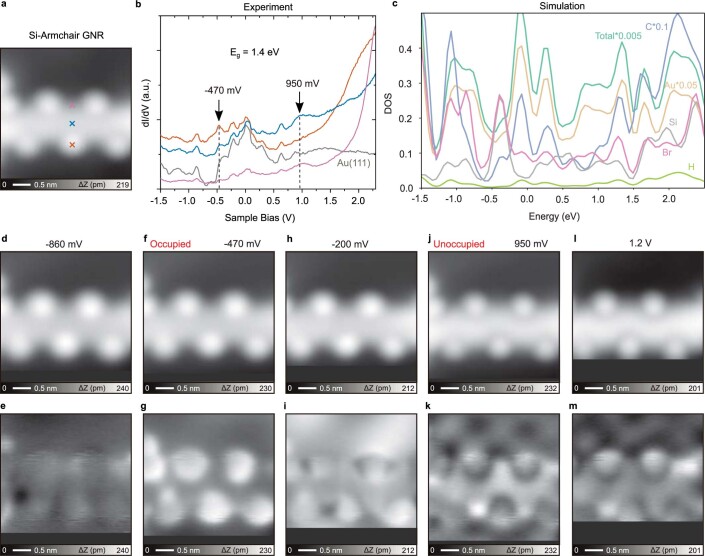
Electronic properties of Si-Armchair GNR. **a**, STM topography of Si-Armchair GNR. **b**, d*I*/d*V* curves measured at the three different sites indicated by crosses in (A) as well as on the clean Au(111) surface as a reference. **c**, Calculated Density of States (DOS). **d**–**m**, A series of STM topographies and the simultaneously recorded d*I*/d*V* maps, measured at different bias voltages. We assigned the occupied and unoccupied states at around −0.47 V and 0.95 V, resulting in a band gap of 1.4 eV. The spatial distribution of occupied and unoccupied states are localized around the Br sites and the carbon backbone, respectively. Measurement parameters: *V* = 200 mV and *I* = 10 pA in (**a**). *V* = −860 mV and *I* = 160 pA in (**d**). *V* = −470 mV and *I* = 160 pA in (**f**). *V* = −200 mV and *I* = 160 pA in (**h**). *V* = 950 mV and *I* = 160 pA in (**j**). *V* = 1.2 V and *I* = 160 pA in (**l**).

After annealing at a higher temperature of 580 K, the structure of oligomers further changed as the bright dots at the edges form a zigzag arrangement along the longitudinal axis (Fig. 4g,h). The high-resolution constant-height d*I*/d*V* image (Fig. 4i) and the corresponding Laplace filtered image (Fig. 4j) show that the C_4_Si_2_ six-membered rings were transformed into the C_4_Si five-membered rings (siloles). The apparent length of C–Si–Br is 3.14 ± 0.20 Å (indicated in Fig. 4i). Unlike the C_4_Si_2_ rings in Si-COF, the C_4_Si_2_ six-membered rings in Si-Cove GNR are not stabilized within the network structure of COF, and thus sequential cyclization from desilicification of the disilabenzene and subsequent dehydrogenation of the pyrene backbones proceed upon thermal activation. Again, the simulated structure and STM images support our analysis (Fig. 4k). The electronic properties of Si-Armchair GNR were investigated by STS measurement and DFT calculations (Extended Data Fig. 8), in which the occupied state peak is at –0.47 V and the unoccupied state peak is at +0.95 V, resulting in a band gap of approximately 1.4 eV. Hence, the high reproducibility of the Si-incorporated COF structure is unambiguously proved.

## Conclusions

In summary, we synthesized 1,4-disilabenzene-bridged COFs by reacting bromo-substituted HBTP molecules and Si atoms on a Au(111) surface. The linked structure of C_4_Si_2_ rings passivated by Br atoms can be determined by bond-resolved STM images combined with DFT calculations as well as XPS measurements. TBP molecules can also form the C_4_Si_2_ rings after reacting with Si atoms and these can then be transformed into C_4_Si rings after desilicification and dehydrogenation. These results demonstrate the high generality of the C–Si on-surface coupling by depositing Si atoms and subsequent polyaromatic hydrocarbons on Au(111). This may further extend the possibilities for syntheses of various low-dimensional nanostructures.

## Methods

### Synthesis of precursors

Materials: All chemicals were purchased from Sigma Aldrich or Kanto Chemicals and were used without further purification unless otherwise described. High-resolution mass spectra (HR-MS) were recorded using a Bruker mirOTOF II with an APCI II module. HBTP was purified by recrystallization from *o*-dichlorobenzene.

Synthesis of 4,5,9,10-tetrabromopyrene (TBP): As per a similar literature procedure^57^, to a 30 ml round-bottom flask, 1,2,3,6,7,8-hexahydropyrene (0.622 g, 3.0 mmol), iron (0.117 g, 2.1 mmol), dichloromethane (20 ml) and bromine (1.70 ml, 33.0 mmol) were added. The reaction mixture was stirred at 45 °C for 23 h. The precipitate was filtered off and washed with acetone and warm chloroform. Recrystallization from *o*-dichlorobenzene afforded white needle-shape crystals (0.391 g, 25%). HR-MS calculated for C_16_H_6_Br_4_: 517.7162, found: 517.7179 (Supplementary Fig. 14).

### Scanning tunnelling microscopy measurement

All the experiments were conducted in a low-temperature scanning tunnelling microscopy (STM) system (home made) at 4.3 K under a high-vacuum environment (<1 × 10^−10^ mbar). The bias voltage was applied to the sample while the tip was electrically grounded. Au(111) surfaces were cleaned through cyclic sputtering (Ar^+^, 10 min) and annealing (720 K, 15 min). Si atoms were deposited on a clean Au(111) surface with an electron beam evaporator (SPECS GmbH). HBTP and TBP molecules were deposited from Knudsen cells (Kentax GmbH). The STM tip was made from chemically etched tungsten. For bond-resolved imaging, the tip apex was terminated by a small CO molecule picked up from the surface^58^. The bias voltage was set close to zero. The modulation amplitude was 7 mV_rms_ and the frequency was 510 Hz.

### Photoemission spectroscopy measurement

Photoemission spectroscopy measurements were conducted on the BL2B beamline at the UVSOR-III Synchrotron, which features a monochromatic light source with a photon energy ranging from 23 to 205 eV. The high-resolution Au 4*f*, Si 2*p* and Br 3*d* spectra were taken with a photon energy of 130 eV, measured in normal emission mode with an overall energy resolution of 0.33 eV. All spectra were processed with a Shirley-background subtraction as the binding energies are respective to the Fermi edge of the Au(111) substrate. The core level spectra fitted by GL(*m*) and SGL(*m*) functions represent the product and sum of Gaussian and Lorentzian functions, respectively. The parameter *m* indicates a ratio between the two functions, as *m* = 0 is a pure Gaussian and *m* = 100 is pure Lorentzian.

### Theoretical calculations

All first-principles calculations on the gold substrate in this work were performed using the periodic plane-wave basis VASP code^59,60^ implementing spin-polarized DFT. To accurately include van der Waals interactions in this system, we used the DFT-D3 method with Becke–Johnson damping^61,62^— various other van der Waals functionals were tested and no significant differences were observed. Projected augmented wave potentials were used to describe the core electrons^63^ with a kinetic energy cutoff of 500 eV (with PREC = accurate). Systematic *k*-point convergence was checked for all systems with sampling chosen according to the system size. This approach converged the total energy of all the systems to the order of 1 meV. The properties of the bulk and surface of Au were carefully checked within this methodology and excellent agreement was achieved with experiments. For calculations of the assemblies on the surface, a vacuum gap of at least 1.5 nm was used. A 3 × 3 × 1 *k*-point grid was used and the upper three layers of Au (five layers in total) and all atoms in the assemblies were allowed to relax to a force of less than 0.01 eV Å^−1^. Atomic structure visualizations were made with the VMD package^64^. Standard simulated STM images were calculated using the CRITIC2 package^65,66^ based on the Tersoff−Hamann approximation^67^. For the high-resolution CO-tip STM images, we have made use of the FHI-AIMS code^68^ with the previous optimized geometry used in a single point calculation. For these calculations the Perdew–Burke–Ernzerhof exchange–correlation functional was used^69^ with a Γ *k*-point only and the standard ‘light’ basis set. The high-resolution CO-tip STM images were then computed by means of the PP-STM code with a fixed tip, where the broadening parameter *η* was set to 0.2 eV (ref. ^70^). The CO tip was approximated by 13% of the signal coming from the *s* orbital and 87% originating from the *p*_*xy*_ orbitals on the probe particle. This gave a good agreement with close approach CO-STM and CO-d*I*/d*V* images^71^. Since for longer range CO-STM images also a 50/50 *s*/*p*_*xy*_ ratio was earlier reported^72^, we show an additional comparison of *s*/*p*_*xy*_ ratios for all the calculated voltage d*I*/d*V* images in Supplementary Fig. 9. DOS analysis was made using the VASPKIT package^73^.

Calculations of the nucleus-independent chemical shift were realized with the ORCA code^74^ at the PBE level. The def2-SZP^75^ basis set was used in all calculations for the lighter atoms. For Au atoms, a zeroth-order regular approximation (ZORA) scalar relativistic Hamiltonian combined with the def2-SVP basis set^76^ and a segmented all-electron relativistically contracted (SARC) basis set combined with the ZORA-TZVP basis set was used. An auxiliary basis set of the type def2/J was introduced to calculate the NMR chemical shifts^77^ in the geometric centre of each ring taken into consideration.

## Online content

Any methods, additional references, Nature Research reporting summaries, source data, extended data, supplementary information, acknowledgements, peer review information; details of author contributions and competing interests; and statements of data and code availability are available at 10.1038/s41557-022-01071-3.

## Supplementary information

Supplementary Information Supplementary Figs. 1–14, Table S1 and references.

## Data Availability

The STM, XPS, DFT and PP-STM computational data and metadata are freely available under a CC BY 4.0 license on the following link: https://zenodo.org/record/6626953
