# Supplementary material for: On-surface synthesis of disilabenzene-bridged covalent organic frameworks
Source: Nat Chem. 2022 Nov 7;15(1):136–42. doi: 10.1038/s41557-022-01071-3 (PMC9836936; doi:10.1038/s41557-022-01071-3)
Supplement: Supplementary file 1 — Supplementary Figs. 1–14, Table S1 and references. [file 41557_2022_1071_MOESM1_ESM.pdf]

nature chemistry

Article

https://doi.org/10.1038/s41557-022-01071-3

# On-surface synthesis of disilabenzene-bridged covalent organic frameworks

In the format provided by the authors and unedited

Supplementary Information for

# On-Surface Synthesis of Disilabenzene-Bridged Covalent Organic Frameworks

Kewei Sun[1], Orlando J. Silveira[2], Yujing Ma[1], Yuri Hasegawa[3], Michio Matsumoto[4], Satoshi Kera[3], Ondřej Krejčí[2], Adam S. Foster*[2,5], Shigeki Kawai*[1,6]

[1]*Research Center for Advanced Measurement and Characterization, National Institute for Materials Science, 1-2-1 Sengen, Tsukuba, Ibaraki 305-0047, Japan*

[2]*Department of Applied Physics, Aalto University, Finland*

[3]*Institute for Molecular Science, Department of Photo-Molecular Science, Myodaiji, Okazaki 444-8585, Japan*

[4]*International Center for Materials Nanoarchitectonics (WPI-MANA), National Institute for Materials Science, 1-1, Namiki, Tsukuba, Ibaraki 305-0044, Japan.*

[5]*WPI Nano Life Science Institute (WPI-NanoLSI), Kanazawa University, Kakuma- machi, Kanazawa 920-1192, Japan*

[6]*Graduate School of Pure and Applied Sciences, University of Tsukuba, Tsukuba 305-8571, Japan*

**Supplementary Figures 1-14**

**Supplementary Table S1**

**References**

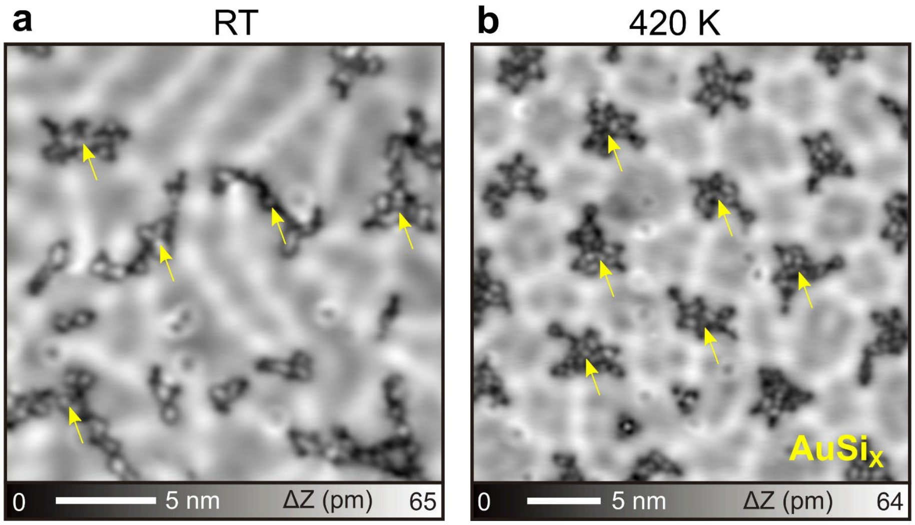


**Supplementary Fig. 1 Depositing Si on Au(111).** (**a**) STM topography of Au(111) after depositing Si atoms kept at room temperature. The contrast of the regions indicated by arrows is darker due to existence of Si atoms. (**b**) STM image of Au(111) covered by Si after annealing at 420 K. The darker regions, whose sizes became more homogeneous, correspond to $AuSi_X$ domains, similar with the previous report[1]. Measurement parameters: Sample bias voltage $V = 100$ mV and tunneling current $I = 100$ pA in (**a**). $V = 100$ mV and $I = 20$ pA in (**b**).

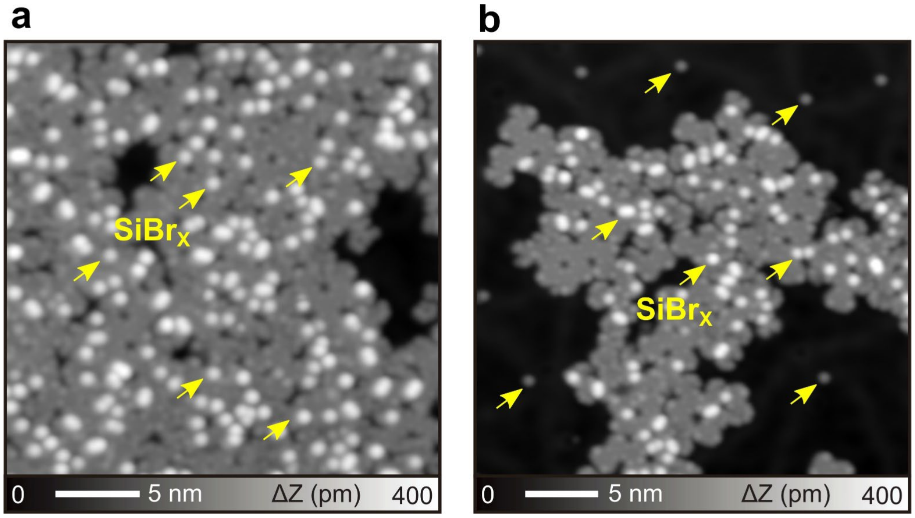


**Supplementary Fig. 2 Nanoporous structures and $SiBr_X$ compounds.** (**a**,**b**) STM topographies of nanoporous structure on Au(111) after heating at 420 K. Many bright dots identified as $SiBr_X$ (X = 1,2,3) compounds were seen on the nanoporous structure and the Au(111) surface as indicated by arrows. Measurement parameters: $V = 200$ mV and $I = 10$ pA in (**a**). $V = 100$ mV and $I = 20$ pA in (**b**).

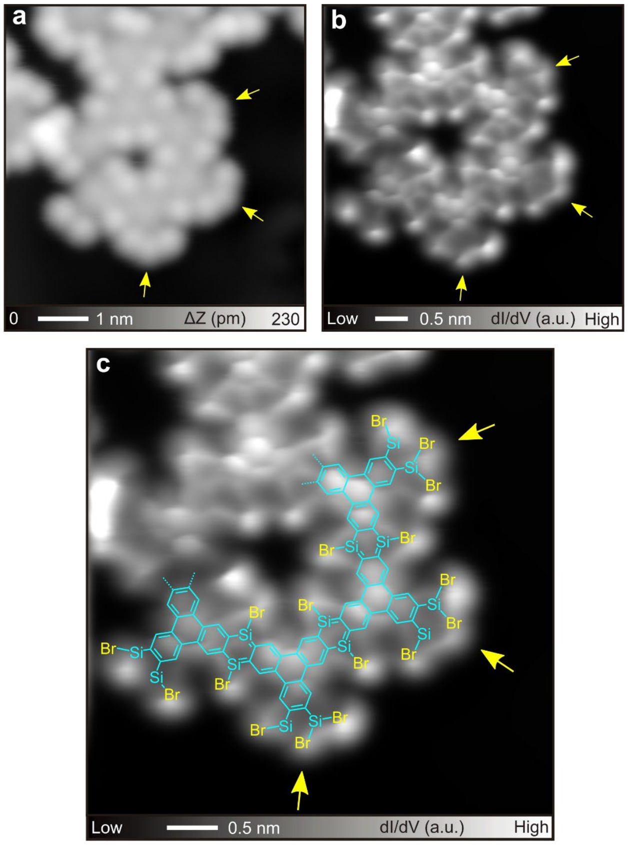


**Supplementary Fig. 3 Si-COF with edges on Au(111).** (**a**) STM topography of single nanoporous structure and (**b**) the corresponding high-resolution constant height d$I$/d$V$ image. (**c**) Chemical structure superimposed on the high-resolution image, which is the same as Supplementary Fig. 3(**b**). The arrows indicate the $SiBr_2$ sites. Measurement parameters: $V$ = 200 mV and $I$ = 5 pA in (**a**).

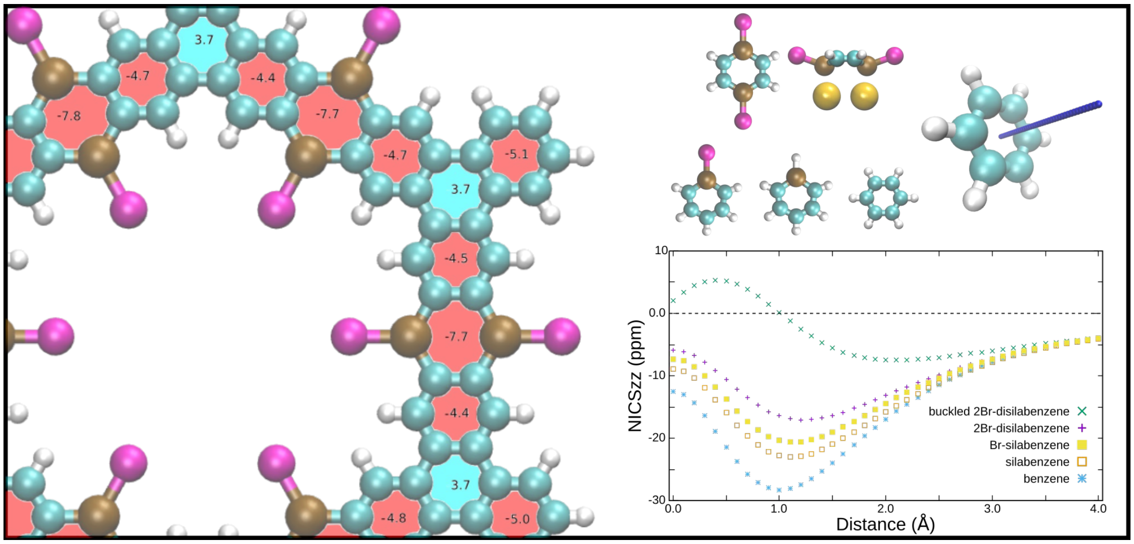


**Supplementary Fig. 4 Calculated NICS results.** On the left, a portion of the proposed COF is shown with its rings coloured in red or blue, representing the aromaticity (red) or antiaromaticity (blue) of the rings. The numbers are the isotropic NICS(0). On the right, we investigated the effect of Au atoms on the NICS. All the rings considered for our simple model are displayed in the top right corner of the image. The buckled 2Br-disilabenzene has 2 Au atoms attached to the bottom of the Si atoms. For benzene, the blue line coming out from its center represents the line where the NICS scan was realized. In the right bottom corner is shown the behavior of the NICSzz component of all rings considered.

We employed DFT calculations to obtain the nucleus independent chemical shift (NICS) in order to investigate the behavior of the π and σ electrons of the COF in the context of nuclear magnetic resonance (NMR) spectroscopy. Considering a planar COF, without the substrate, the isotropic NICS shown in Supplementary Fig. 4 reveal that the 2Br-disilbenzene rings are aromatic, and their aromaticity is slightly stronger than the aromaticity of their neighboring rings. Since the aromaticity measured by the values of the NICS is in fact a magnetic aromaticity, which means that it depends on the magnetic field generated by the moving electrons on the system, the NICS value may vary strongly if the rings are deposited on a metallic substrate, for example, and may give unrealistic results concerning the behavior of π electrons. To eliminate this problem in our calculations, we considered a simplified model for the calculation of the NICS to investigate the aromaticity of the buckled disilabenzene rings that compose our proposed COF. We first took an

isolated planar 2Br-disilabenzene passivated with hydrogens with a planar geometry, and then two Au atoms were considered exactly beneath the Si atoms, distorting the ring in a similar fashion to that observed in our proposed COF. We considered first the zz component of the NICS matrix, which gives a more realistic view of the magnetic fields generated by the aromatic electrons in the z direction[2]. The plot shown in Supplementary Fig. 4 reveals the zz component of the NICS of several different compounds. The *x* axis of the plot references height above the ring in the case of flat rings, or a plane defined by the Si atoms for the buckled structure with attached Au atoms. Our calculations reveal, as expected, that all standard rings such as benzene and silabenzene have a negative NICS starting from the geometrical center, and it peaks around 1 Å for all cases. For the buckled ring, however, the behavior is rather different. The zz component of the NICS is positive until 0.8 Å, which matches with the height of the Br atoms, and then it is negative, peaking around 1.8 Å. This behavior is expected in the vicinity of the buckled ring, since the σ electrons in the ring also generate a magnetic field, but it then decays much faster than the magnetic field from the π electrons, hence the effect observed after 0.8 Å.

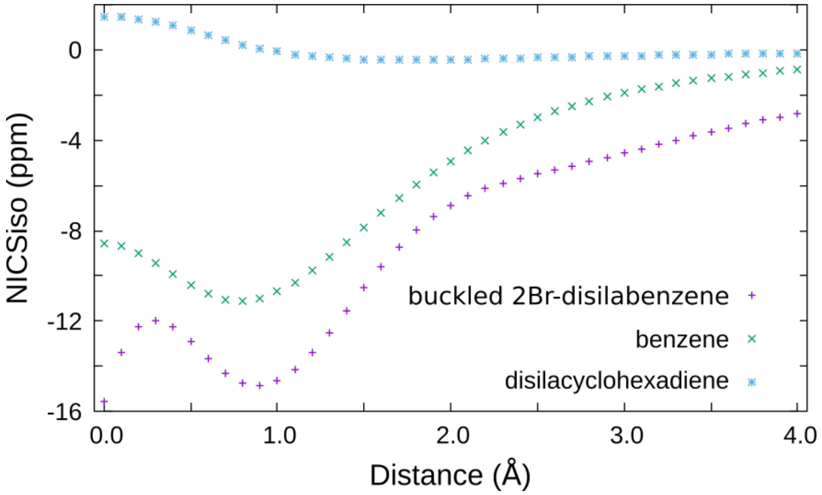


**Supplementary Fig. 5 NICS values as a function of distance of buckled 2Br-disilabenzene, benzene and disilacyclohexadiene.**

Supplementary Fig. 5 shows that the isotropic NICS curve of the buckled 2Br-disilabenzene, where the two Au atoms are considered in the calculation, is typical for an aromatic system, aside from the small deviation in the plot when the probe is "inside" the ring between 0.0 and 0.8 Å. For comparison, the same plot for benzene (aromatic) and disilacyclohexadiene (non-aromatic) are shown, reinforcing the aromatic characteristic of the buckled 2Br-disilabenzene that composes the COF proposed in this work.

**Supplementary Table 1**: Isotropic NICS(0) and NICS(1)

| | benzene | silabenzene | Br-silabenzene | 2Br-disilabenzene | 2Br-buckled-disilabenzene |
|---|---|---|---|---|---|
| NICS(0) | -8.562 | -7.978 | -8.363 | -8.142 | -14.727 |
| NICS(1) | -10.704 | -8.965 | -8.688 | -7.775 | -7.987 |

Supplementary Table 1 depicts the isotropic NICS(0) and NICS(1) calculated for all the rings considered in Supplementary Fig. 4. For the buckled ring, the 0 position was considered to be the plane formed by the Br atoms, and the 1 position is 1 Å above. Results show that buckled ring is indeed aromatic, even with the Au atoms below the Si.

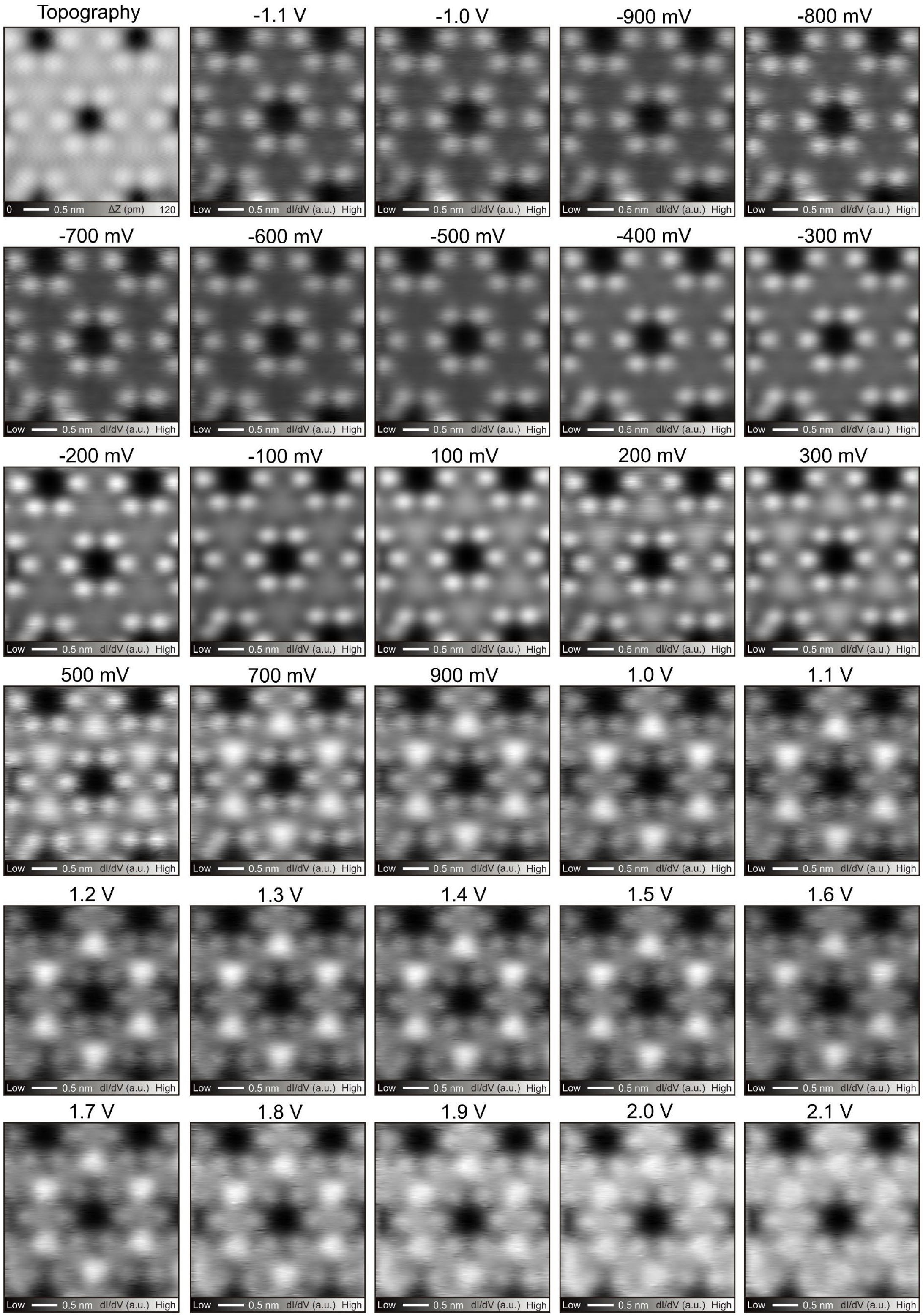


**Supplementary Fig. 6 Electronic properties of Si-COF.** STM topography of Si-COF ($V$ = 200 mV and $I$ = 10 pA) and a series of constant height d$I$/d$V$ maps for Si-COF measured with a CO-tip at different bias voltages.

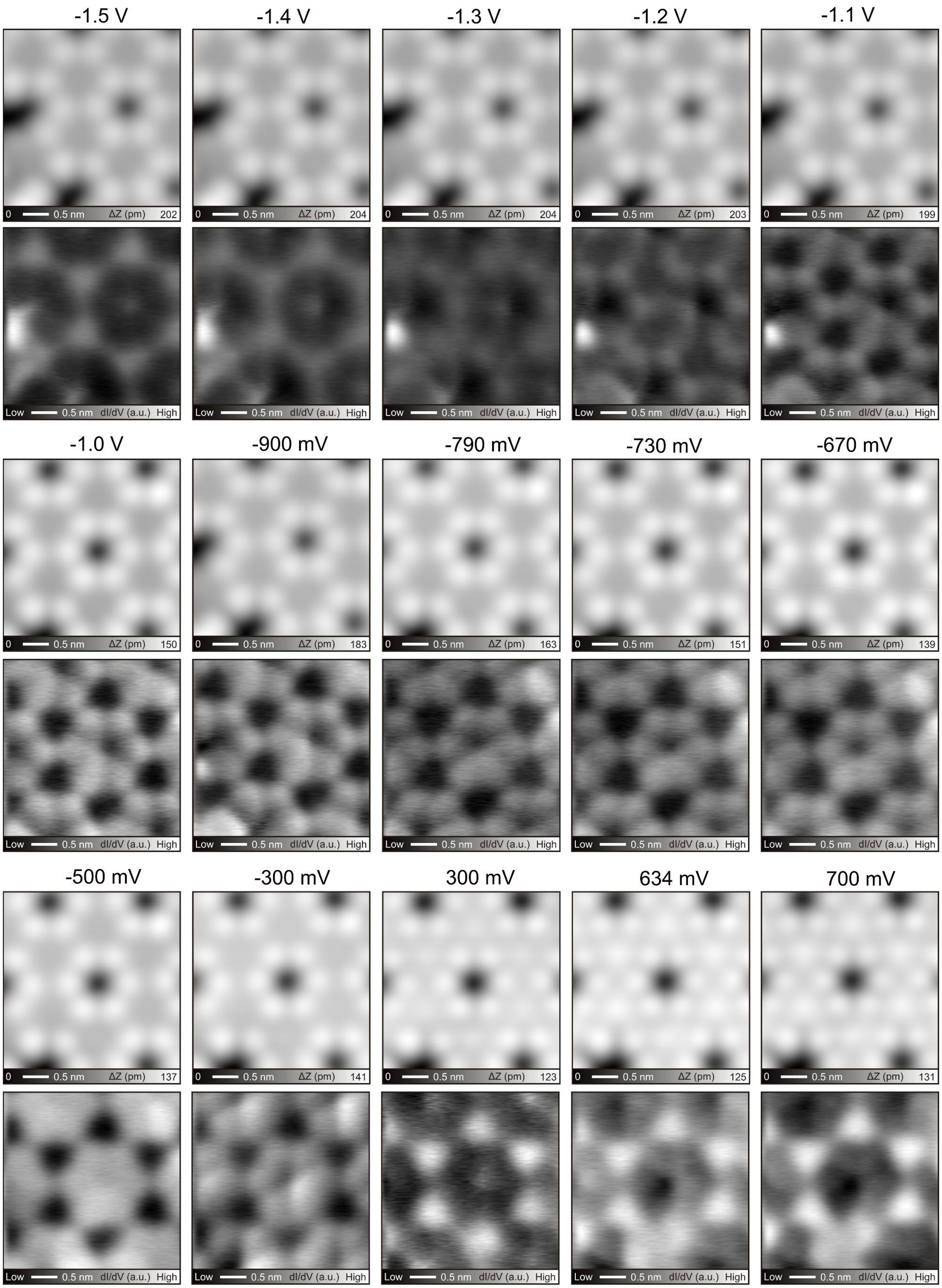

-1.5 V
-1.4 V
-1.3 V
-1.2 V
-1.1 V
0 0.5 nm ΔZ (pm) 202
0 0.5 nm ΔZ (pm) 204
0 0.5 nm ΔZ (pm) 204
0 0.5 nm ΔZ (pm) 203
0 0.5 nm ΔZ (pm) 199
Low 0.5 nm dI/dV (a.u.) High
-1.0 V
-900 mV
-790 mV
-730 mV
-670 mV
0 0.5 nm ΔZ (pm) 150
0 0.5 nm ΔZ (pm) 183
0 0.5 nm ΔZ (pm) 163
0 0.5 nm ΔZ (pm) 151
0 0.5 nm ΔZ (pm) 139
-500 mV
-300 mV
300 mV
634 mV
700 mV
0 0.5 nm ΔZ (pm) 137
0 0.5 nm ΔZ (pm) 141
0 0.5 nm ΔZ (pm) 123
0 0.5 nm ΔZ (pm) 125
0 0.5 nm ΔZ (pm) 131

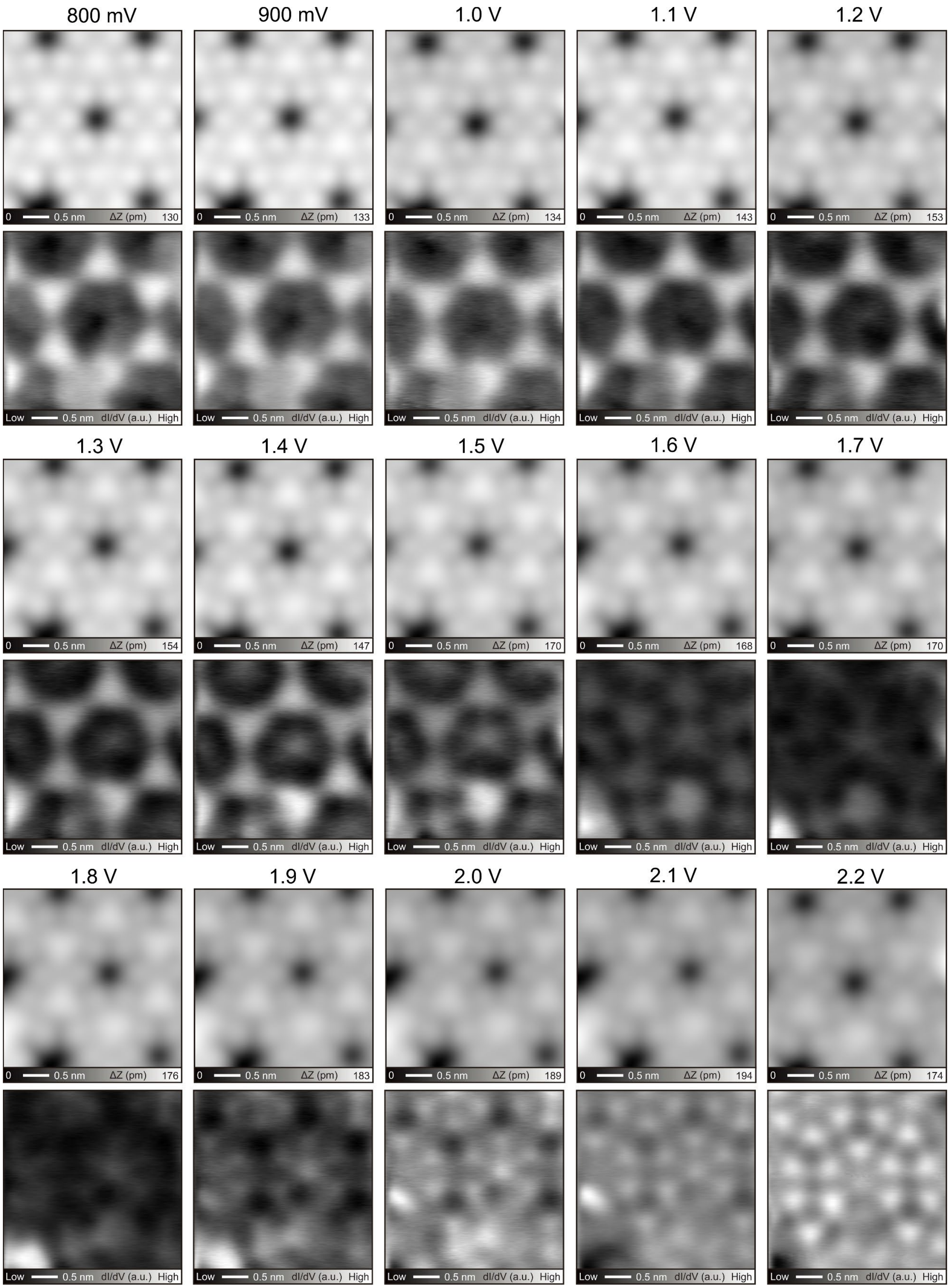


**Supplementary Fig. 7 Electronic properties of Si-COF.** A series of STM topographies of Si-COF and the corresponding constant current d$I$/d$V$ maps measured at different bias voltages.

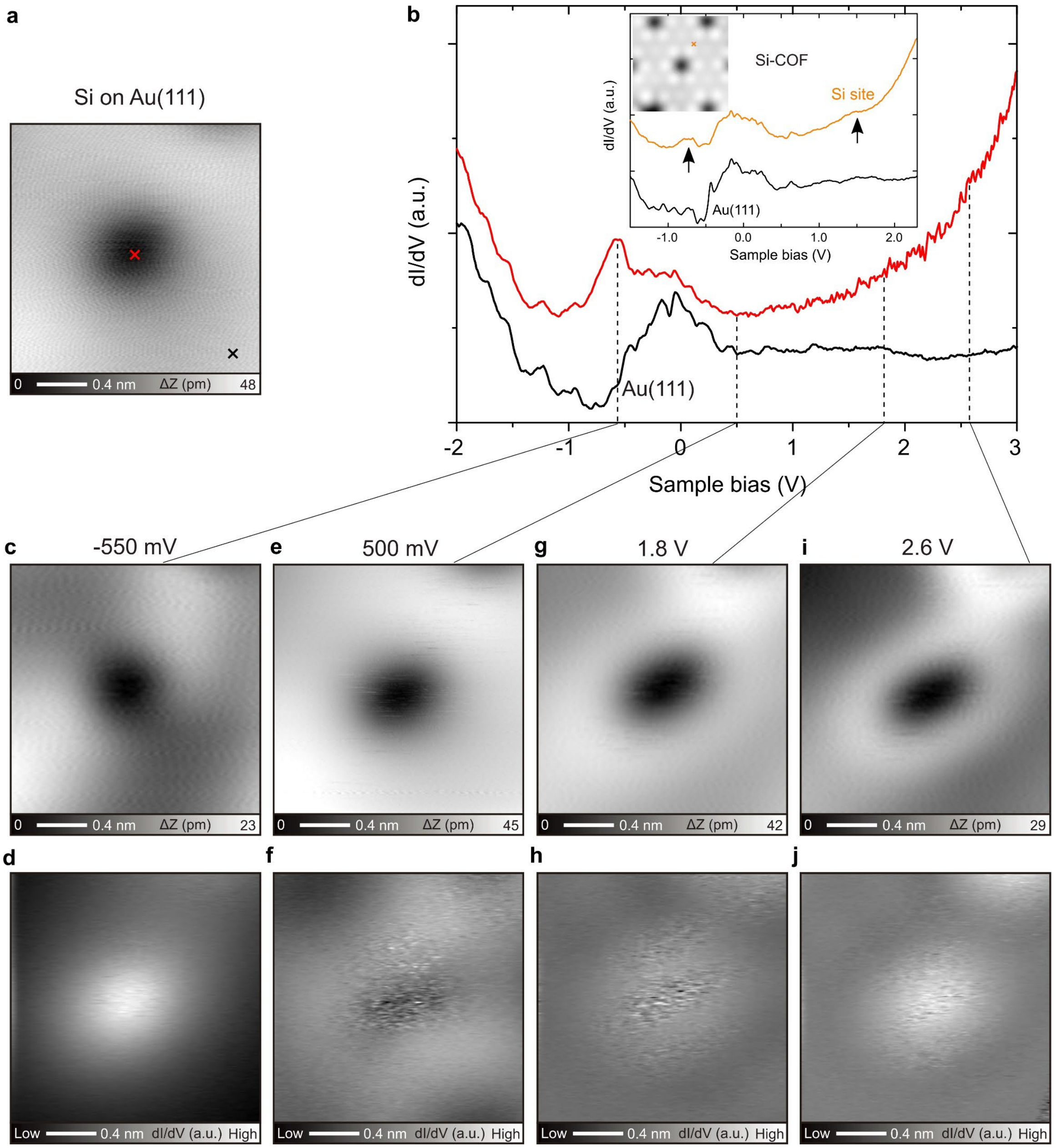


**Supplementary Fig. 8 Electronic properties of single Si on Au(111) and Si in Si-COF.** (**a**) STM topography of one Si on Au(111). (**b**) d*I*/d*V* curves recorded above Si atom and Au(111) surface in (**a**). Inset shows the d*I*/d*V* curves recorded above Si site in Si-COF and bare Au(111) surface. (**c**-**j**)STM topographies and the corresponding constant current d*I*/d*V* maps of single Si on Au(111) with different bias voltages. Measurement parameters: Sample bias voltage $V = 200$ mV and tunneling current $I = 10$ pA in (**a**).

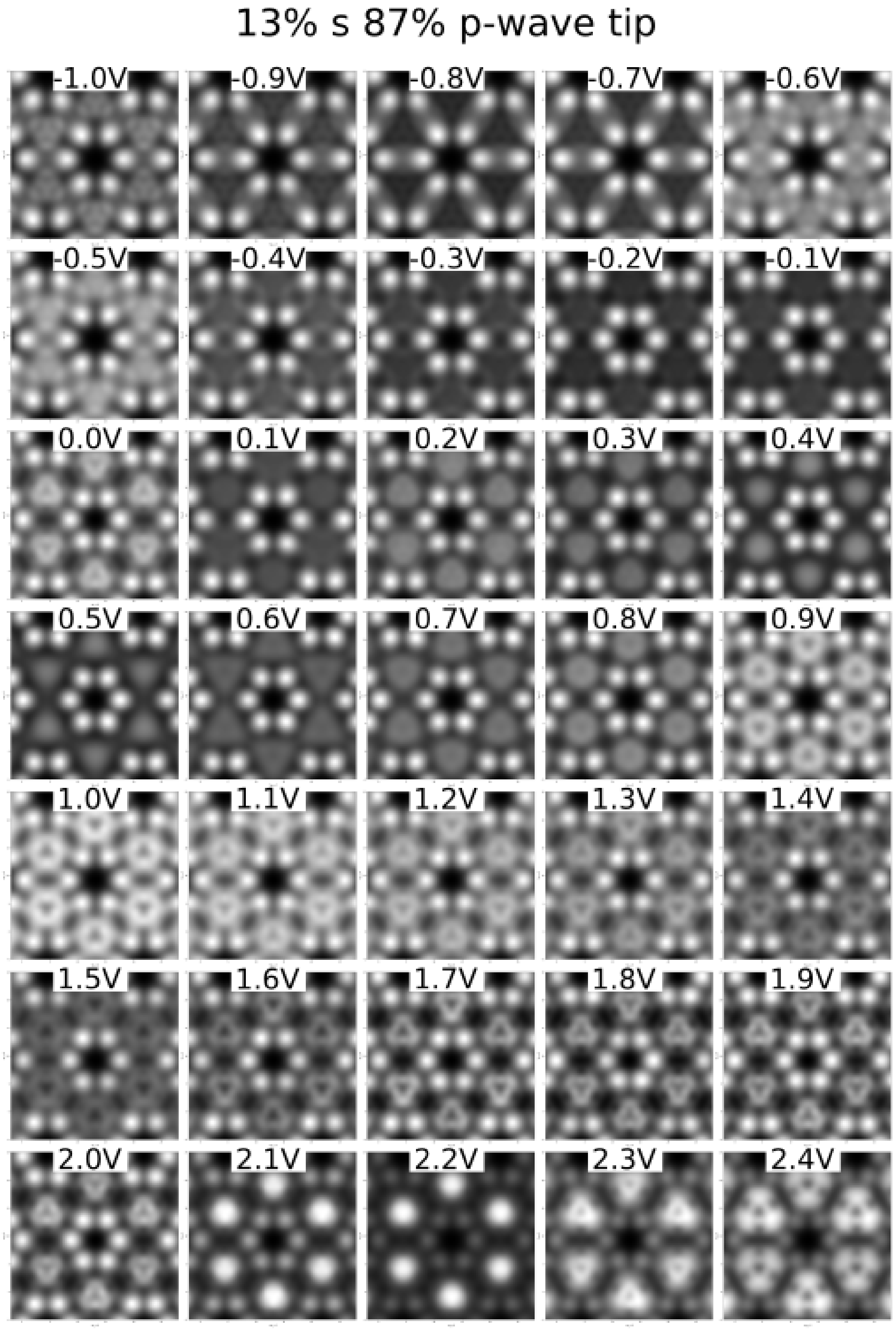
13% s 87% p-wave tip
-1.0V
-0.9V
-0.8V
-0.7V
-0.6V
-0.5V
-0.4V
-0.3V
-0.2V
-0.1V
0.0V
0.1V
0.2V
0.3V
0.4V
0.5V
0.6V
0.7V
0.8V
0.9V
1.0V
1.1V
1.2V
1.3V
1.4V
1.5V
1.6V
1.7V
1.8V
1.9V
2.0V
2.1V
2.2V
2.3V
2.4V

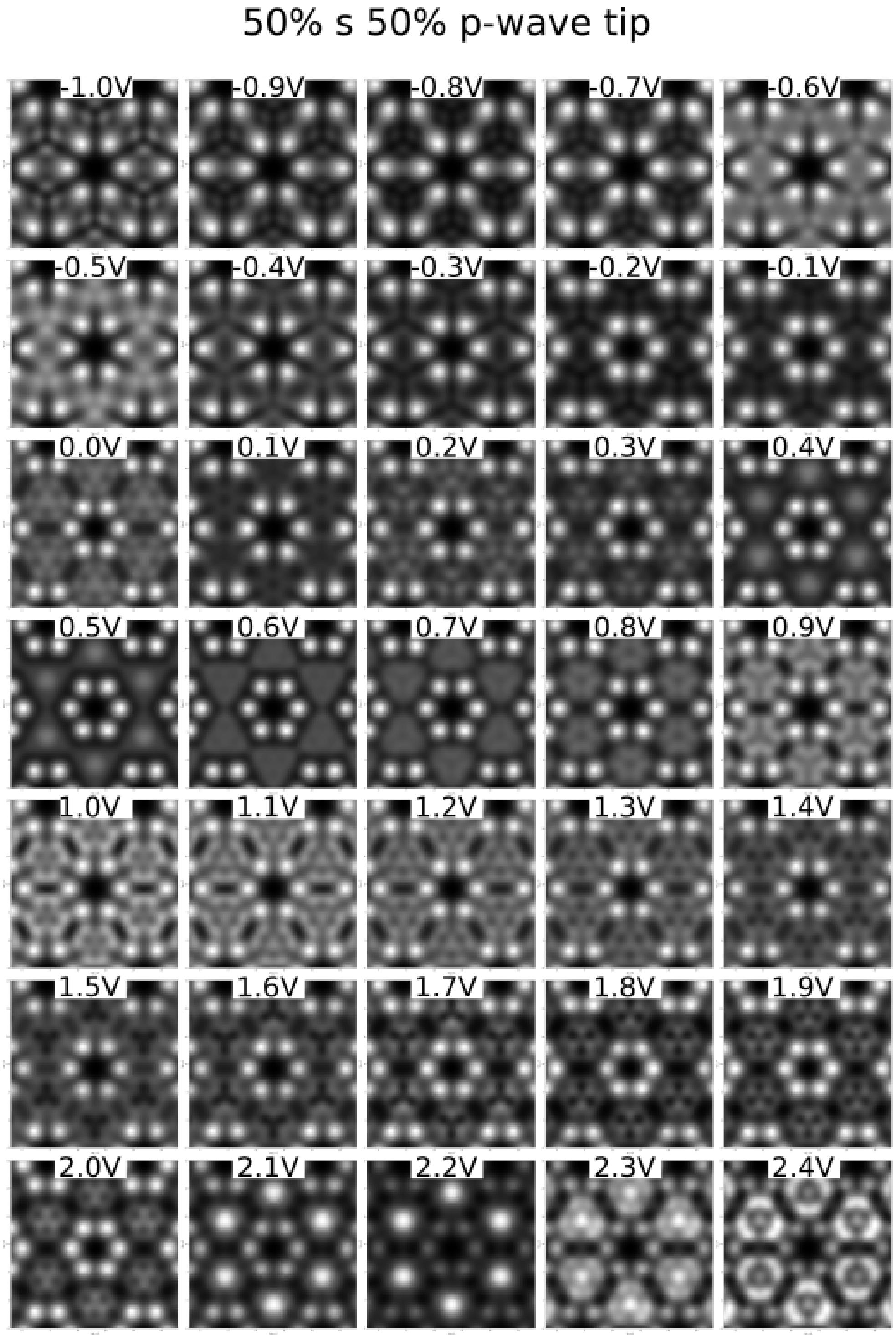


**Supplementary Fig. 9 Calculated d*I*/d*V* maps**. A series of d*I*/d*V* constant height simulations obtained with mixed *s* and *p*-wave tips in order to simulate the CO tip in the experiment. The images were obtained through a linear combination of *p* ($p_x$ and $p_y$) and s orbitals, where we considered the portions shown in the top of the images.

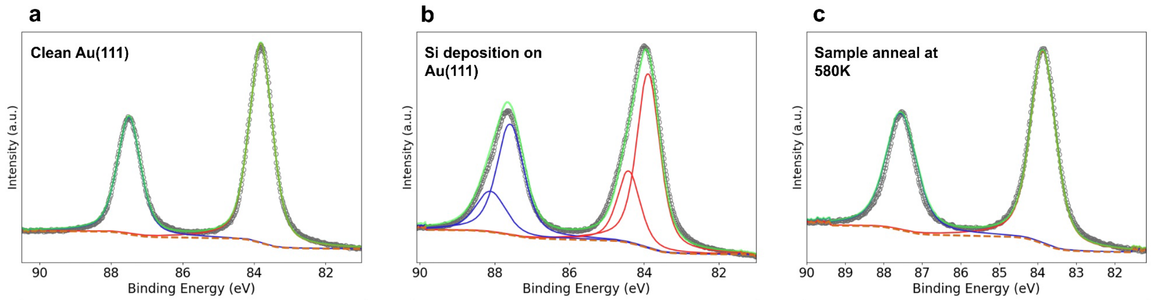


| Name | Position | FWHM | Line shape | Area % |
|---|---|---|---|---|
| Au $4f_{7/2}$ | 83.8045 | 0.76698 | SGL(55) | 42.86 |
| Au $4f_{5/2}$ | 87.4921 | 0.79776 | SGL(55) | 57.14 |

| Name | Position | FWHM | Line shape | Area % |
|---|---|---|---|---|
| Au $4f_{7/2}$ | 83.8903 | 0.8 | SGL(55) T(7) | 40.60 |
| Au $4f_{5/2}$ | 87.5779 | 0.832 | SGL(55) T(7) | 30.45 |
| Au $4f_{7/2}$ ($AuSi_x$) | 84.4047 | 0.9 | SGL(55) T(7) | 16.54 |
| Au $4f_{5/2}$ ($AuSi_x$) | 88.0923 | 0.936 | SGL(55) T(7) | 12.41 |

| Name | Position | FWHM | Line shape | Area % |
|---|---|---|---|---|
| Au $4f_{7/2}$ | 83.8515 | 0.77 | SGL(55) T(7) | 57.14 |
| Au $4f_{5/2}$ | 87.5391 | 0.95 | SGL(55) T(7) | 42.86 |

**Supplementary Fig. 10 High-resolution XPS Au 4f spectra taken after each experimental step.** (**a**) Well-defined Au 4f spectra with doublet peaks separated by 3.69 eV. (**b**) After Si deposited on Au substrate, the Au 4f becomes broaden significantly. Two sets of doublets are following the same peak separation as well as the intensity ratio. (**c**) When the Si-COF is formed upon annealing at 580K, the Au 4f transformed back to the doublet peaks that are quite comparable with the clean surface situation.

Detailed fitting parameters of all XPS spectra are described in a table underneath each plot. Fittings followed general protocols, such as doublet peaks associated with various orbitals adopt well-defined energy separations. The Au 4f doublet components $4f_{7/2}$ and $4f_{5/2}$ have a large energy separation of 3.69 eV; Si 2p doublet components $2p_{3/2}$ and $2p_{1/2}$ are slightly separated by 0.61 eV in binding energy; and Br 3d doublet, $3d_{5/2}$ and $3d_{3/2}$ has an intermediate energy separation of 1.05 eV. The other restraints applied in spectra fitting are the area (intensity) ratios between the doublet components, where Au 4f doublet has an intensity ratio of 0.75 ($4f_{7/2} : 4f_{5/2} = 4:3$); Si 2p doublet has an intensity ratio of 0.5 ($2p_{3/2} : 2p_{1/2} = 2:1$); Br 3d doublet adopted the intensity ratio of 0.67 ($3d_{5/2} : 3d_{3/2} = 3:2$).

Our best fitting of the Au 4f spectra was well-reproduced with a line shape of SGL(55)T(7), which represent a Gaussian/Lorentzian sum form with a slight underlying asymmetric profile blend T(k). Si 2p doublet peaks are fitted with line shape of GL(30), it represents a Gaussian/Lorentzian product form. Detailed mathematical equations are available in CasaXPS library. In each spectra plot, the raw XPS data and convoluted

envelope obtained by fitting are indicated by hollow circle markers and semi-transparent green lines, respectively. Each individual component is indicated by different colored lines, where each doublet set is plotted with red and blue curves, and the Shirley background is indicated with brown dash lines.

Si atoms were deposited on a clean Au(111) surface with an electron beam evaporator (SPECS GmbH). HBTP were deposited from a Knudsen cells (Kentax GmbH). The deposition and annealing parameters were the same as those in the STM measurement.

In Supplementary Fig. 10b, upon Si deposition onto the Au substrate, a discernable broadening of each Au 4f doublet peaks indicates the presence of two chemical components. The second set of doublet peaks were shifted by 0.51 eV to high binding energy compared to the bulk gold. Shifting to a higher BE for the gold-silicide ($AuSi_X$) alloy on the Au(111) was commonly reported. However, one might find it a bit contradictory considering the significant Si-Au interaction mentioned in the main text, yet both Au 4f and Si 2p spectra shift to the higher BE side. In fact, this phenomenon was well explained before and proposed with a d-electron depletion model[3]. It is understood that on the account of Au-Si interaction, Si transfers s charge to Au, but Au loses more localized d-electrons forming a s-d hybrid bond, in which the key factor is that the Coulomb interaction between Au 4f and the 6s conduction electron is quite different from that of 4f and 5d interaction, with the latter one actually significantly larger (by ~3eV) and affecting the overall binding states of Au.

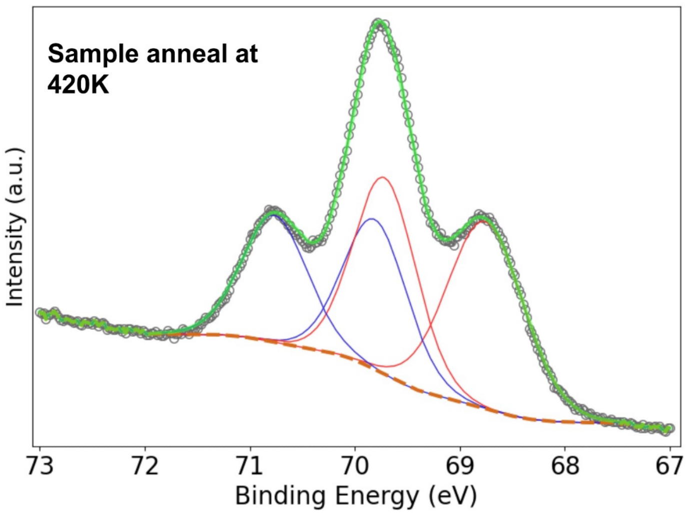


| Name | Position | FWHM | Line shape | Area % |
|---|---|---|---|---|
| Br $3d_{5/2}$ | 68.7558 | 0.85091 | GL(30) | 31.70 |
| Br $3d_{3/2}$ | 69.8058 | 0.71902 | GL(30) | 21.13 |
| Br $3d_{5/2}$ -2 | 69.7149 | 0.70599 | GL(30) | 28.30 |
| Br $3d_{3/2}$ -2 | 70.7649 | 0.73875 | GL(30) | 18.87 |

**Supplementary Fig. 11 High-resolution XPS Br 3d spectra at Si-COF formation after depositing HBTP precursor molecule at 420 K.** The doublet sets are best fitted with a separation of 1.05 eV and an intensity ratio of 0.67, and color coded as red and blue sets. Two sets of the doublet indicated two major types of Br-species at the intermediate stage, which possibly corresponds to disassociated $Br_2$ on the surface and Si-Br bonded species.

In Supplementary Fig. 11, the lower binding energy set (Br $3d_{5/2}$ locate at 68.76 eV) were assigned to disassociated Br atoms/clusters[4] and higher BE position ($3d_{5/2}$ locate at 69.71 eV) was in good agreement with previous studies[5-7]. Compared to the C-Br bonding related Br 3d energy positions in these reports, our component at higher BE is highly accountable for Si-Br bond forming a silicon tetrabromide ($SiBr_4$) compound which can be easily desorbed with higher temperature annealing. In addition, it is worth mentioning that the bromine signal essentially vanished after annealing at high temperature of 580 K, which is possibly due to different experimental setups (e.g. higher annealing temperature) at beamline.

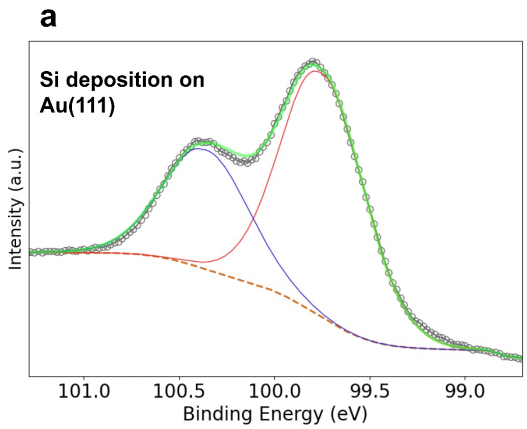


| Name | Position | FWHM | Line shape | Area % |
|---|---|---|---|---|
| Si $2p_{3/2}$ | 99.7543 | 0.5071 | GL(30)T(8) | 66.67 |
| Si $2p_{1/2}$ | 100.366 | 0.5153 | GL(30)T(8) | 33.33 |

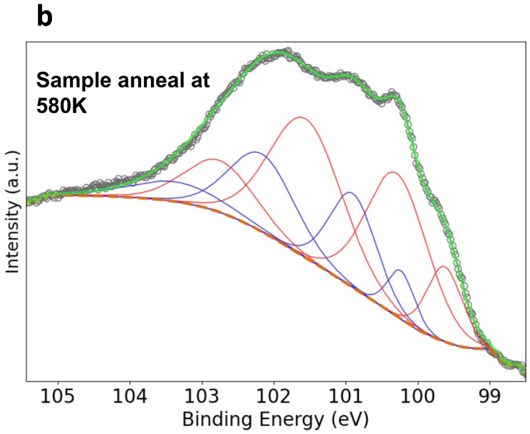


| Name | Position | FWHM | Line shape | Area % |
|---|---|---|---|---|
| Si $2p_{3/2}$ | 99.6207 | 0.60849 | GL(30) | 8.00 |
| Si $2p_{1/2}$ | 100.231 | 0.46302 | GL(30) | 4.00 |
| Si $2p_{3/2}$-2 | 100.327 | 1.03555 | GL(30) | 24.04 |
| Si $2p_{1/2}$-2 | 100.937 | 0.78309 | GL(30) | 12.02 |
| Si $2p_{3/2}$-3 | 101.623 | 1.18989 | GL(30) | 26.53 |
| Si $2p_{1/2}$-3 | 102.233 | 1.11913 | GL(30) | 13.27 |
| Si $2p_{3/2}$-4 | 102.803 | 1.17288 | GL(30) | 8.09 |
| Si $2p_{1/2}$-4 | 103.413 | 1.5 | GL(30) | 4.05 |

**Supplementary Fig. 12 High-resolution XPS Si 2p spectra after each experimental step.** (**a**) The as-deposited Si on Au substrate shows well-defined doublet peaks with a slight shift to higher binding energy compare to that of bulk silicon peak positions. We attribute this to the formation of $AuSi_X$, as supported by the Au 4f spectra at that experimental step. (**b**) Upon formation of Si-incorporated COF structure, the Si 2p has a shape that best fits with a minimum of 4 sets of doublets components, where predominantly two relatively large areas indicate a majority of two types of Si-species exist.

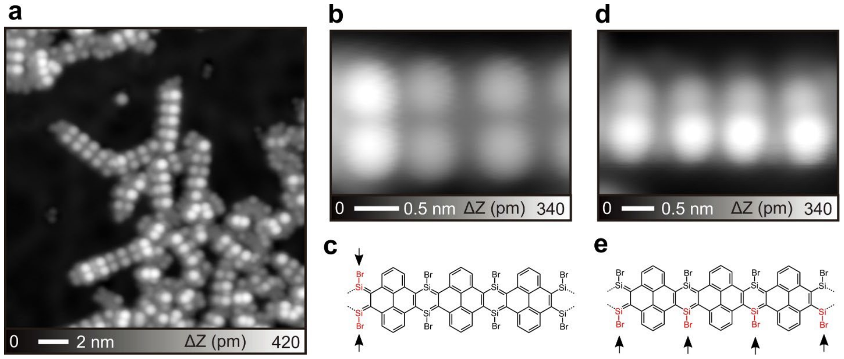


**Supplementary Fig. 13 Si-Cove GNRs on Au(111).** (**a**) STM topography of Si-Cove GNRs on Au(111) after heating at 420 K. (**b**) Closeup view of Si-Cove GNR with brighter dots at the left-hand. The brighter contrast relates to a higher adsorption height of the Br atom (Si-Br). (**c**) Corresponding chemical structure, in which the bright contrast parts were described in the red color. Red Si-Br indicated by arrows means the higher position from surface. (**d**) Another example, in which the Br atoms at the lower sites are higher than those at the upper sites, appeared brighter as shown in (**e**). Measurement parameters: $V$ = 100 mV and $I$ = 5 pA in (**a**). $V$ = 200 mV and $I$ = 5 pA in (**b**,**c**).

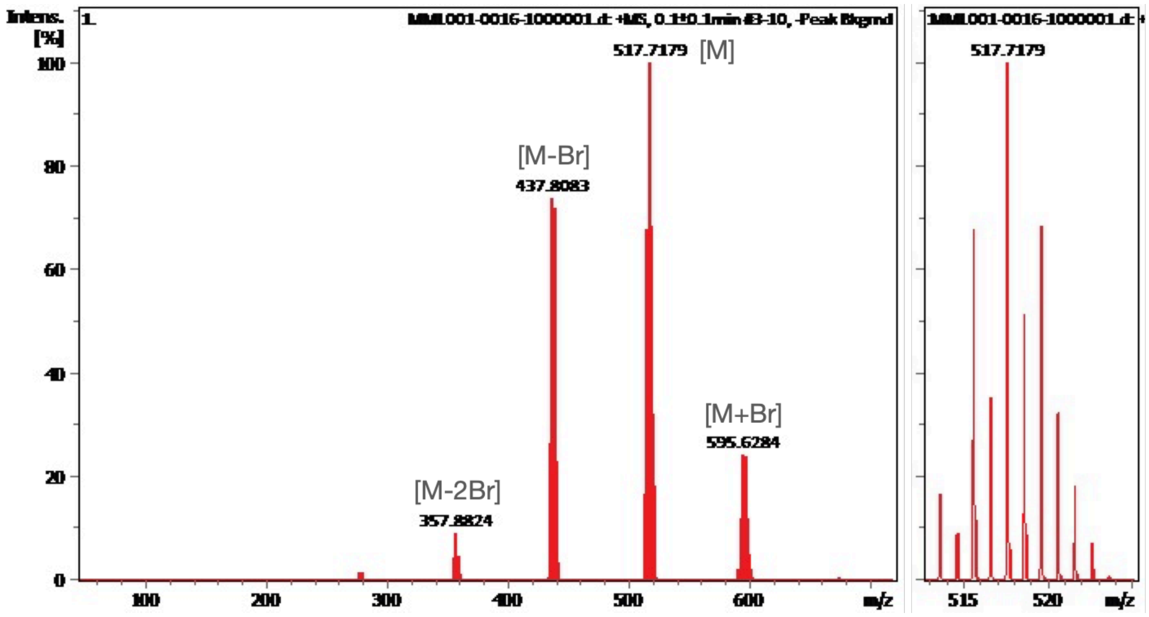


**Supplementary Fig. 14 High-resolution mass spectra of TBP.** (Left) Wide range spectrum and (right) magnified range spectrum.

**References**


1. Sun, K., Nishiuchi, T., Sahara, K., Kubo, T., Foster, A. S. & Kawai, S. Low-temperature removal of dissociated bromine by silicon atoms for an on-surface Ullmann reaction. *J. Phys. Chem. C* **124**, 19675−19680 (2020).
2. Stanger, A. Nucleus-independent chemical shifts (NICS): distance dependence and revised criteria for aromaticity and antiaromaticity. *J. Org. Chem.* **71**, 883-893 (2006).
3. Watson, R. E., Hudis, J. & Perlman, M. L. Charge flow and d compensation in gold alloys. *Phys. Rev. B* **4**, 4139–4144 (1971).
4. Jankovský, O., Šimek, P., Klimová, K., Sedmidubský, D., Matejková, S., Pumerac, M. & Sofer, Z. Towards graphene bromide: bromination of graphite oxide. *Nanoscale* **6**, 6065–6074 (2014).
5. Smykalla, L., Shukrynau, P., Korb, M., Lang, H. & Hietschold, M. Surface-confined 2D polymerization of a brominated copper-tetraphenylporphyrin on Au(111). *Nanoscale* **7**, 4234–4241 (2015).
6. Cardenas, L., Gutzler, R., Lipton-Duffin, J., Fu, C., Brusso, J. L., Dinca, L. E., Vondráček, M., Fagot-Revurat, Y., Malterre, D., Rosei, F. & Perepichka, D. F. Synthesis and electronic structure of a two dimensional π-conjugated polythiophene. *Chem. Sci.* **4**, 3263–3268 (2013).
7. Eichhorn, J., Strunskus, T., Rastgoo-Lahrood, A., Samanta, D., Schmittele, M. & Lackinger, M. On-surface Ullmann polymerization via intermediate organometallic networks on Ag(111). *Chem. Commun.* **50**, 7680–7682 (2014).
